# Changes in the airway microbiome in patients with bronchiectasis

**DOI:** 10.1097/MD.0000000000036519

**Published:** 2023-12-15

**Authors:** Dongmei Lu, Chenxi Li, Zhiwei Zhong, Maidina Abudouaini, Aynazar Amar, Hongtao Wu, Xuemei Wei

**Affiliations:** a Division of Respiratory and Critical Care Medicine, People’s Hospital of Xinjiang Uygur Autonomous Region, Urumqi, Xinjiang Uygur Autonomous Region, China; b Department of Oral and Maxillofacial Oncology Surgery, the First Affiliated Hospital of Xinjiang Medical University, School/Hospital of Stomatology, Xinjiang Medical University, Stomatological Research Institute of Xinjiang Uygur Autonomous Region, Urumqi, Xinjiang Uygur Autonomous Region, China; c Graduate School, Xinjiang Medical University, Urumqi, Xinjiang Uygur Autonomous Region, China.

**Keywords:** acute exacerbation, bronchiectasis, bronchoalveolar lavage fluid, metagenomic next-generation sequencing

## Abstract

This study used metagenomic next-generation sequencing (mNGS) technology to explore the changes of the microbial characteristics in the lower respiratory tract in patients with acute exacerbations of bronchiectasis (noncystic fibrosis) to guide clinical treatment and improve patients’ quality of life and prognosis. This prospective study included 54 patients with acute exacerbation and 46 clinically stable patients admitted to the Respiratory and Critical Care Medicine Center of the People’s Hospital of Xinjiang Uygur Autonomous Region from January 2020 to July 2022. Sputum was subjected to routine microbiological tests, and bronchoalveolar lavage fluid (BALF) samples were subjected to microbiological tests and mNGS of BALF before empirical antibiotic therapy. Serum inflammatory markers (white blood cell count, interleukin-6, procalcitonin, and C-reactive protein) were measured. In addition, we evaluated the pathogen of mNGS and compared the airway microbiome composition of patients with acute exacerbation and control patients. The mean age of our cohort was 56 ± 15.2 years. Eighty-nine patients had positive results by mNGS. There was a significant difference in the detection of viruses between the groups (χ^2^ = 6.954, *P* < .01). The fungal species *Candida albicans, Pneumocystis jirovecii*, and *Aspergillus fumigatus* were significantly more common in patients with acute exacerbations (χ^2^ = 5.98, *P* = .014). The bacterial species *Acinetobacter baumannii, Mycobacterium tuberculosis, Haemophilus influenzae, Haemophilus parahaemolyticus, Abiotrophia defectiva,* and *Micromonas micros* were significantly more prevalent in patients with acute exacerbations (χ^2^ = 4.065, *P* = .044). The most common bacterial species isolated from the sputum and BALF samples of patients with acute exacerbation was *A. baumannii. Chlamydia psittaci* was found in 4 patients. In addition, of 77 patients with negative sputum culture, 66 had positive results by mNGS, demonstrating the increased sensitivity and accuracy of mNGS. Patients with acute exacerbation of bronchiectasis tend to have mixed infections in the lower respiratory tract. The frequency of viruses, fungi, and *Mycoplasma* was higher in these patients. Our findings suggest that mNGS could be used to identify pathogenic microorganisms in these patients, increasing the effectiveness of antibiotic therapy.

## 1. Introduction

Bronchiectasis is a chronic airway disease with multifactorial etiology. Disease prevalence is increasing globally but varies by race and geographical region.^[1,2]^ The prevalence of bronchiectasis in the United States and Europe is 701 and 1200 per 100,000.^[3,4]^ Nonetheless, little is known about the epidemiology of bronchiectasis in China.

The estimated prevalence of laboratory-diagnosed bronchiectasis (noncystic fibrosis [non-CF]) in adults (age ≥ 40 years) in China from 2002 to 2004 was 1.2%.^[5]^ The causes of bronchiectasis include primary ciliary dyskinesia, gastroesophageal reflux, allergic bronchopulmonary aspergillosis, systemic connective tissue diseases (rheumatoid arthritis, Sjogren syndrome, and scleroderma), cystic pulmonary fibrosis, bacterial and fungal infections (tuberculosis, nontuberculous mycobacterial infection, and aspergillosis), and viral infections, leading to airway inflammation, airway wall destruction, and bronchiectasis. The mechanisms underlying bronchiectasis are unclear, and the lack of effective treatments affects patients’ health and quality of life. Even patients without acute exacerbation have symptoms. The detection rate of bacteria can reach 60%,^[6]^ which is closely related to the prognosis of patients with acute exacerbation and quality of life.

Few studies measured the prevalence of mycobacteria, fungi, and viruses in patients with bronchiectasis. In addition, the prevalence of *Staphylococcus aureus* in these patients is underestimated. The rate of detection and accuracy of identification of pathogenic bacteria in sputum samples are low.^[7]^ The influence of the airway microbiota on the occurrence and development of bronchiectasis is unclear. It is difficult to measure the diversity of airway bacteria using traditional analytical methods. The composition of the microbiome in the respiratory tract of patients with bronchiectasis can be analyzed by metagenomics. Little is known about the changes in the composition of microbial communities during disease progression. Moreover, the results of changes in airway microbiota composition in patients with acute exacerbation of bronchiectasis are contradictory. It is plausible that, given differences in genetics, environment, and lifestyle, the airway microbiome may vary across different geographical regions and differ from ethnic groups.^[8]^ It is imperative to reveal these factors affecting the distribution on the community structure of the respiratory tract microbiota.

The aim of this study is to analyze the changes of airway microbial diversity and inflammatory factors during stable and acute exacerbation bronchiectasis in different ethnic groups by metagenomic next-generation sequencing (mNGS). This study may potentially provide insight into the pathogenic characteristics of the disease, optimize the individualized selection of antibiotics from a new perspective, and provide a new entry point for the intervention of airway infection and destruction. It may also potentially provide an important theoretical basis for guiding us to take targeted prevention, control and treatment measures, and reduce the number of hospitalizations for acute exacerbated bronchiectasis.

## 2. Materials and methods

### 2.1. Study subjects

The study included 54 patients with acute exacerbation of bronchiectasis and 46 clinically stable patients (controls) admitted to the Respiratory and Critical Medicine Center of the People’s Hospital of Xinjiang Uygur Autonomous Region from January 2020 to July 2022. Bronchiectasis (non-CF) was defined by radiological confirmation of bronchiectasis by either dynamic computed tomography (CT) or high-resolution CT thorax in accordance with British Thoracic Society guidelines along with the absence of any other major concurrent chronic respiratory disease state. The inclusion criteria were: patients with a history of bronchiectasis or bronchiectasis diagnosed using chest high-resolution CT; age ≥ 18 years; a history of stable bronchiectasis or a history of antibiotic use within 4 weeks of recruitment; and patients with acute exacerbation, defined as aggravation of respiratory symptoms over 24 hours, including cough, dyspnea, expectoration, or purulent sputum with or without hemoptysis, chest pain, fever, and abnormal lung sounds. The exclusion criteria were: patients with other respiratory diseases; patients with severe immunosuppression, patients who have taken systemic glucocorticoids or immunosuppressants within 3 months; absence of microbial culture results; poor compliance and unable to cooperate with clinical observation and specimen collection; and loss to follow-up.

The study protocol was approved by the Research Ethics Committee of People’s Hospital of Xinjiang Uygur Autonomous Region. Patients who met inclusion criteria will be enrolled after obtaining signed informed consent.

Changes in airway microbiome abundance and diversity in patients with and without acute exacerbation were compared.

### 2.2. Clinical data collection

Demographic characteristics and associated clinical information were collected, including age, sex, ethnicity, body mass index, smoking history, lung function, serum levels of inflammatory markers (interleukin-6, procalcitonin, and C-reactive protein), and CT images, from all patients.

### 2.3. Specimen collection and processing

Sputum samples were subjected to routine microbiological tests, and bronchoalveolar lavage fluid (BALF) samples were subjected to microbiological tests and mNGS. Sputum collection conducted by a specialist in the Center of Respiratory and Critical Care Medicine of Xinjiang Uygur Autonomous Region. Sputum samples were collected on the day of patients’ admission and were delivered to the special microbiological testing laboratory. BALF samples were collected at the center of intervention by bronchoscopy under local anesthesia by physicians with more than 5 years of clinical experience. Airway topical anesthesia was performed using 2% lidocaine. The bronchoscope was introduced through the nose and advanced to segmental bronchi of the affected lung, and 60 to 120 mL of warm saline (37ºC) was injected for lavage. At least 10 mL of BALF was collected into a sterile container at a negative pressure of 100 mm Hg and divided into 2 aliquots. One aliquot was subjected to bacterial and fungal culture and acid-fast staining for *Mycobacterium tuberculosis*. The other aliquot was sent to Beijing Genomics Institute for mNGS. Sputum samples (at least 1 mL) were collected by patient-initiated cough in a sterile container and subjected to bacterial and fungal culture and acid-fast staining.

DNA extraction and reverse transcription, library construction and sequencing, and data processing and analysis were performed as described previously. Briefly, 3 to 5 mL of BALF was centrifuged at 4000 rpm for 10 minutes at 4°C. Pellet DNA was extracted using the TIANamp Micro DNA Kit [DP316, Tiangen Biotech, Beijing, China] according to the manufacturer’s instructions. The extracted DNA was fragmented by sonication to a size of 200 to 300 bp and quantified using the Qubit dsDNA HS Assay Kit on an Agilent 2100 Bioanalyzer (Agilent Technologies Inc, Santa Clara, California, USA). Circular single-stranded DNA underwent cyclization to produce DNA nanoballs by rolling circle amplification. DNA nanoballs were loaded into the sequencing chip and sequenced using the BGISEQ-50/MGISEQ-2000 platform. Low-quality bases (quality score < 20) and short fragments (<35 bp) were removed from raw reads. Reads mapped to the human reference genome using BWA (http://bio-bwa.sourceforge.net/) were removed. The obtained sequences were searched against databases of bacteria, viruses, fungi, and parasites.

### 2.4. Ethics statement

This study was approved by the Research Ethics Committee of the People’s Hospital of Xinjiang Uygur Autonomous Region. All patients or their legal guardians gave written informed consent.

### 2.5. Statistical analysis

Statistical analysis was performed using SPSS Statistics version 24.0 and R version 4.1.2. Continuous variables were compared using an independent-samples *t* test and expressed as mean ± standard deviation or median (interquartile range). Categorical variables were compared using the χ^2^ test and represented as frequencies or percentages. Variables were compared by univariate analysis and logistic regression analysis. All tests were 2-sided. *P* values <0.05 were considered statistically significant.

## 3. Results

**Figure 1.**
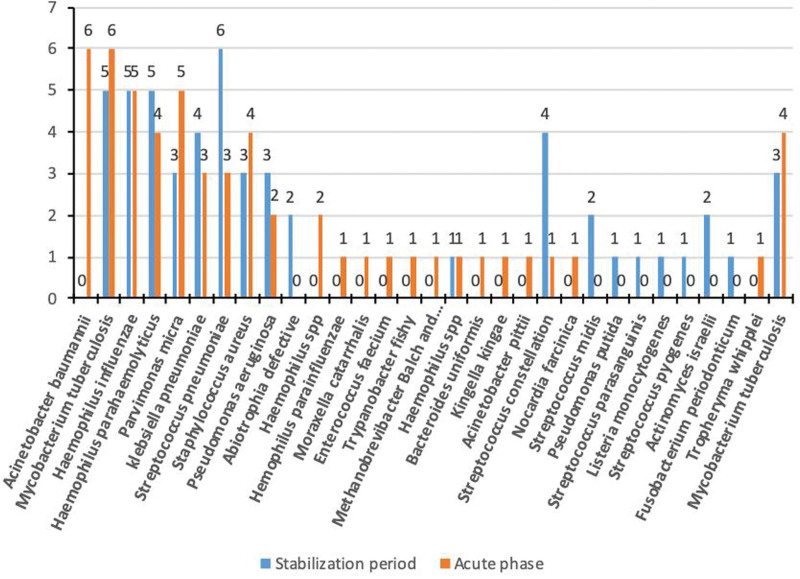
Frequency of detection of bacterial species by metagenomic next-generation sequencing in patients with and without acute exacerbation of bronchiectasis.

**Figure 2.**
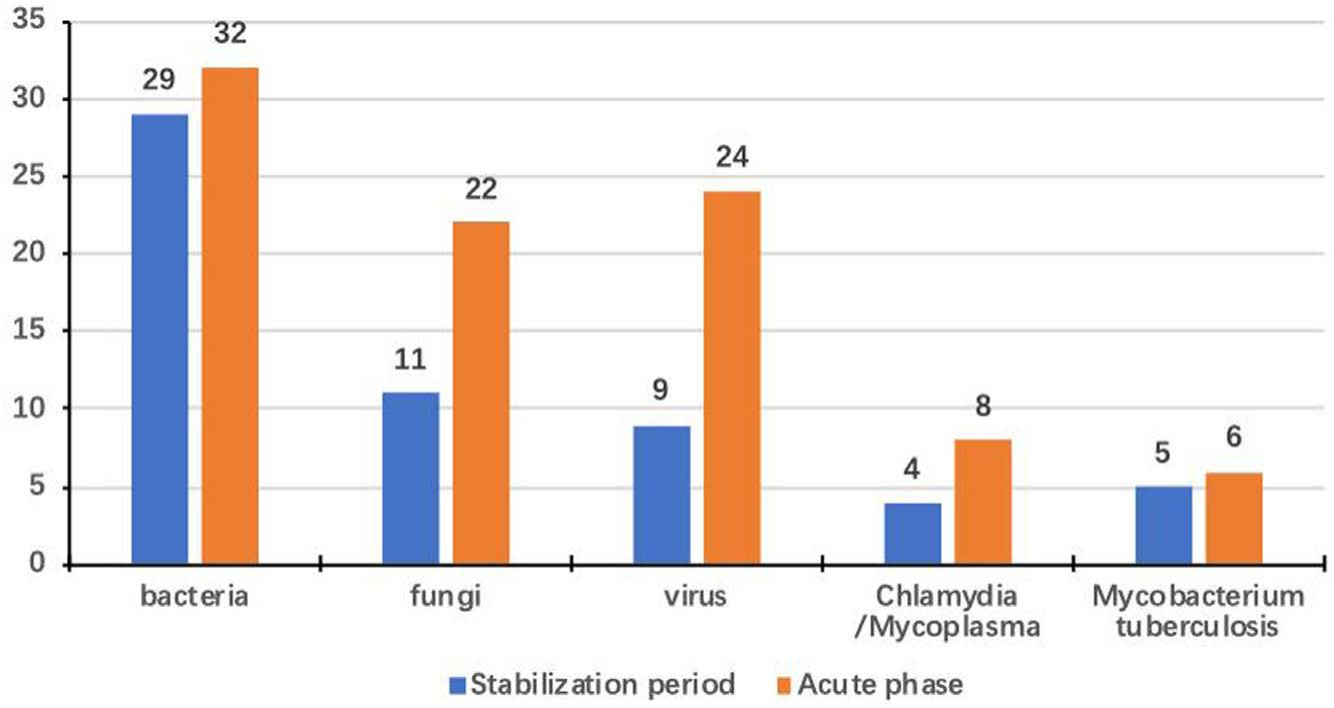
Frequency of detection of pathogens by metagenomic next-generation sequencing in patients with and without acute exacerbation of bronchiectasis.

**Figure 3.**
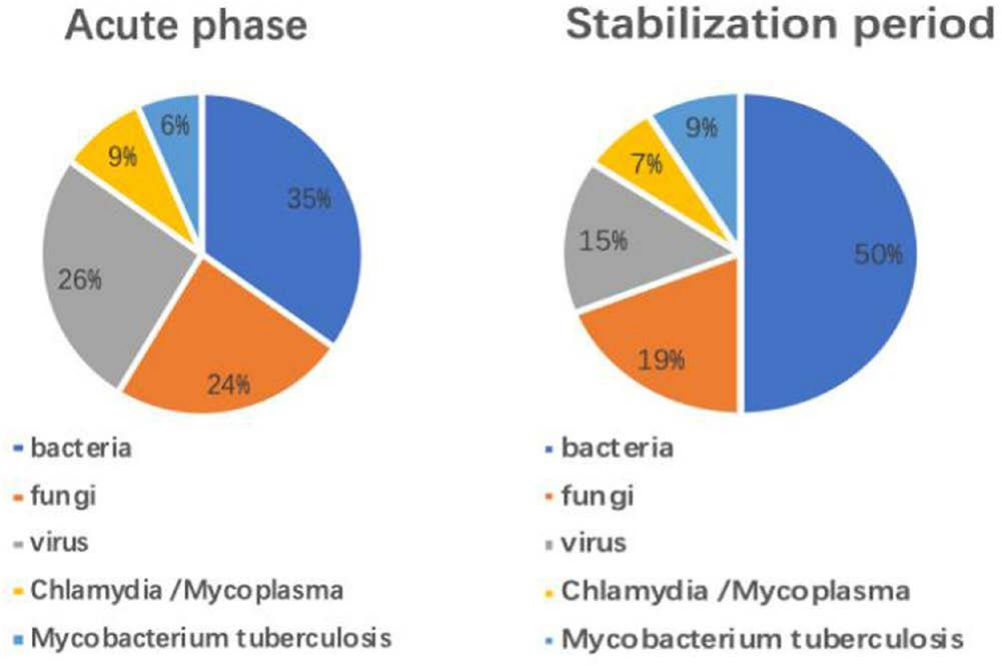
Frequency of detection of *Mycobacterium tuberculosis* and other pathogens by metagenomic next-generation sequencing in patients with and without acute exacerbation of bronchiectasis.

The average age of our cohort (51 males and 49 females) was 56.0 ± 15.2 years (range, 23–82 years). Of 100 BALF samples, 89 were positive by mNGS. There was no significant difference in age, gender, and ethnicity between the study groups. Fifty-four microorganisms were identified. The prevalence of bacterial species (31), fungal species (10), and viruses (10) is shown in Table 1 and Figures 1, 2, and 3. *Mycoplasma* and *Chlamydia psittaci* were found in 11 and 4 samples, respectively. The most commonly isolated fungal species were *Candida albicans* (11/100), *Pneumocystis jirovecii* (11/100), and *Aspergillus fumigatus* (6/100). Of 100 sputum samples, 77 were culture-negative and 23 were culture-positive (Table 2). Eleven microorganisms were identified in our samples. Bacterial species, viruses, fungal species, and *Mycoplasma* were found in 17, 3, 2, and 1 sample, respectively. The most commonly detected bacteria were *M. tuberculosis* (6/100), *Acinetobacter baumannii* (4/100), *Klebsiella pneumoniae* (4/100), *Legionella pneumophila* (2/100), *S. aureus* (1/100), and *Escherichia coli* (1/100). Influenza A and B viruses were detected in 2 and 1 sample, respectively. *Aspergillus* and *Mycoplasma pneumoniae* were found in 1 sample each.

**Table 1:** Frequency of detection of pathogens in the bronchoalveolar lavage fluid of patients with and without acute exacerbation of bronchiectasis.

Pathogen	Acute exacerbation patients	Clinically stable patients	χ^2^	*P value*
Bacteria				
* *Positive cases	32 (59.26)	29 (63.04)	0.15	.699
* Acinetobacter baumannii*	6 (11.11)	0 (0.00)	5.44	.02
* Mycobacterium tuberculosis*	6 (11.11)	5 (10.87)	0.001	.969
* Haemophilus influenzae*	5 (10.87)	5 (9.26)	0.072	.789
* Haemophilus parahaemolyticus*	5 (10.87)	3 (5.56)	0.953	.329
* Abiotrophia defectiva*	2 (3.70)	0 (0.00)	1.738	.187
* Parvimonas micra*	5 (9.26)	3 (6.52)	0.253	.615
* Klebsiella pneumoniae*	4 (7.41)	3 (6.52)	0.03	.863
* Staphylococcus aureus*	4 (7.41)	3 (6.52)	0.03	.863
* Pseudomonas aeruginosa*	2 (3.70)	3 (6.52)	0.415	.519
* Streptococcus pneumonia*	3 (5.56)	6 (13.04)	1.701	.192
* Constellation constellatus*	1 (1.85)	5 (10.87)	3.52	.058
* Streptococcus mitis*	0 (0.00)	2 (2.00)	2.36	.122
Viruses				
Positive cases	24 (44.44)	9 (19.57)	6.954	.008
* *Human γ herpesvirus type 4	16 (29.63)	3 (6.52)	8.619	.003
* *Human β herpesvirus type 7	7 (12.96)	5 (10.87)	0.103	.748
* *Human α herpesvirus type 1	6 (11.11)	0 (0.00)	5.437	.020
* *Human β herpesvirus type 6A	1 (1.85)	2 (4.35)	0.532	.466
* *Human β herpesvirus type 5	3 (5.56)	1 (2.17)	0.740	.390
* Torque teno virus*	1 (1.85)	1 (2.17)	0.013	.909
* *Human herpes virus 8	1 (1.85)	0 (0.00)	0.860	.354
* * β papillomavirus type 2	1 (1.85)	0 (0.00)	0.860	.354
* Torque teno virus* 10	0 (0.00)	1 (2.17)	1.186	.276
* Torque teno virus* 15	0 (0.00)	1 (2.17)	1.186	.276
Fungus				
Positive cases	11 (23.91)	22 (40.74)	3.181	.074
* Pneumocystis jirovecii*	7 (12.96)	4 (8.70)	0.462	.497
* Candida albicans*	8 (14.81)	3 (5.56)	3.024	.082
* Aspergillus fumigatus*	5 (9.26)	1 (2.17)	2.211	.137
* Candida glabrum*	2 (3.70)	0 (0.00)	1.738	.187
* Aspergillus versicolor*	1 (1.85)	1 (2.17)	0.013	.909
*Aspergillus terreus*	1 (1.85)	0 (0.00)	0.86	.354
* Aspergillus*	1 (1.85)	0 (0.00)	0.86	.354
* Candida parapsilosis*	0 (0.00)	2 (4.35)	2.396	.122
* Pneumocystis jirovecii*	0 (0.00)	1 (2.17)	1.186	.276
* Malassezia globosa*	0 (0.00)	1 (2.17)	1.186	.276
*Chlamydia/Mycoplasma*				
Positive cases	12 (22.22)	4 (8.70)	3.382	.066
* Mycoplasma* spp.	7 (12.96)	4 (8.70)	0.462	.497
* Ureaplasma parvum*	1 (1.85)	0 (0.00)	0.086	.354
* Chlamydia psittaci*	4 (7.41)	0 (0.00)	3.549	.06

Data are numbers and percentages.

**Table 2:** Frequency of detection of pathogens in the sputum of patients with acute exacerbation of bronchiectasis and control patients.

Pathogen	Acute exacerbation patients	Clinically stable patients	χ^2^	*P value*
Bacteria				
Positive cases	12 (22.22)	4 (8.70)	3.382	.066
* Acinetobacter baumannii*	4 (7.41)	0 (0.00)	3.549	.06
* Mycobacterium tuberculosis*	4 (7.41)	2 (4.35)	0.412	.521
* Klebsiella pneumoniae*	3 (5.56)	1 (2.17)	0.74	.39
* Legionella pneumophila*	1 (1.85)	1 (2.17)	0.013	.909
* Escherichia coli*	1 (1.85)	0 (0.00)	0.86	.354
* Streptococcus pyogenes*	0 (0.00)	1 (2.17)	1.186	.276
* Staphylococcus aureus*	1 (1.85)	0 (0.00)	0.86	.354
Viruses				
Positive cases	2 (3.70)	1 (2.17)	0.2	.665
* *Influenza A virus	1 (1.85)	1 (2.17)	0.013	.909
* *Influenza B virus	1 (1.85)	0 (0.00)	0.86	.354
Fungus				
* *Positive cases	1 (1.85)	1 (2.17)	0.013	.909
* Aspergillus*	1 (2.17)	0 (0.00)	0.86	.354
* *Unknown fungus	0 (0.00)	1 (1.85)	1.186	.276
*Mycoplasma*				
Positive cases	0 (0.00)	1 (2.17)	1.186	.276
* Mycoplasma pneumoniae*	0 (0.00)	1 (2.17)	1.186	.276

Data are numbers and percentages.

The prevalence of viruses, fungi, and *Mycoplasma* was higher in patients with acute exacerbation. The prevalence of viruses in patients with and without acute exacerbation was 24% and 9% (χ^2^ = 6.954). The frequency of fungal species was significantly higher in patients with acute exacerbation (20% vs 8%) (χ^2^ = 5.98, *P* = .014). The prevalence of mycoplasma was marginally higher in patients with acute exacerbation (7% vs 4%) (χ^2^ = 0.462, *P* = .048). The frequency of bacterial species was significantly higher in patients with acute exacerbation (29% vs 17%) (χ^2^ = 4.065, *P* = .044). There were no significant differences in the frequency of pathogens in sputum samples between the 2 groups (Table 2).

**Figure 4.**
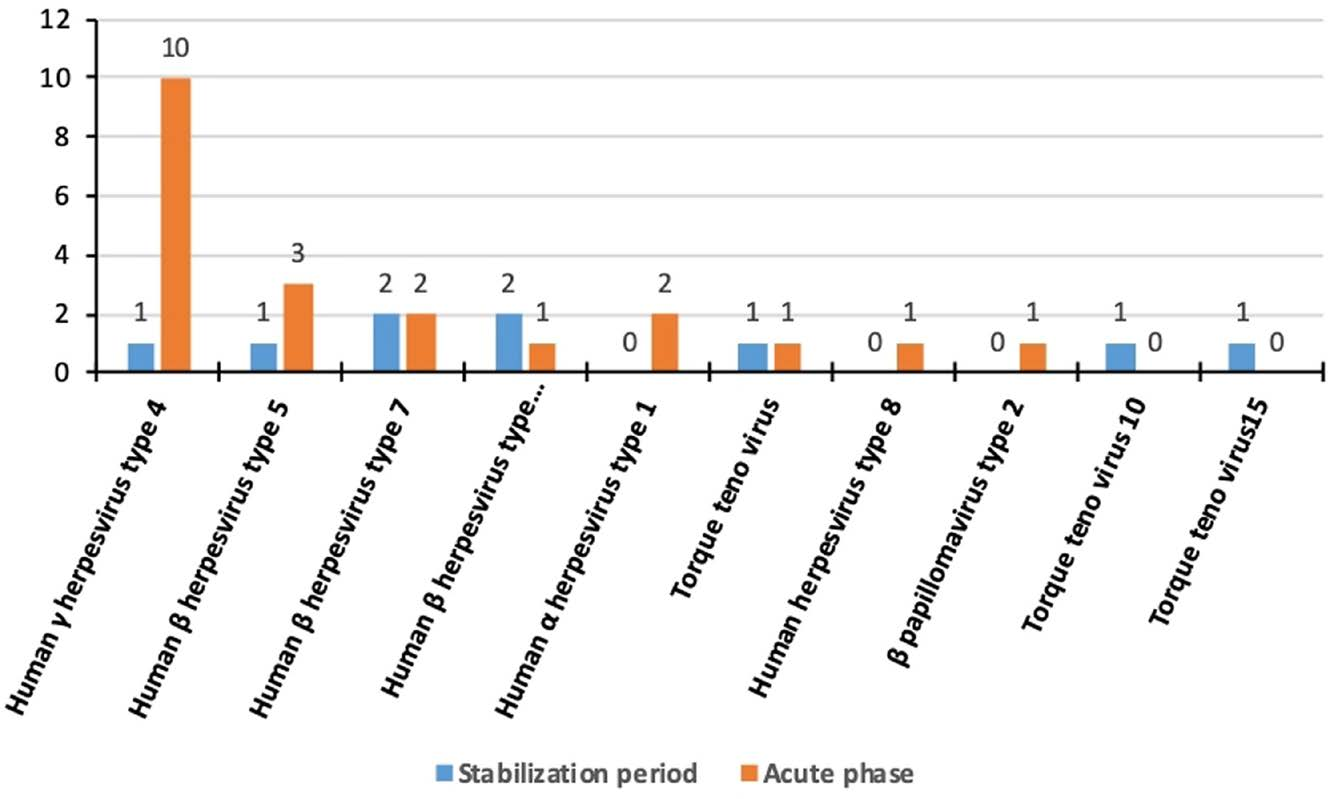
Frequency of detection of viruses by metagenomic next-generation sequencing in patients with and without acute exacerbation.

The 10 most frequent bacterial species in BALF samples from patients with acute exacerbation were *A. baumannii* (6/100), *M. tuberculosis* (6/100), *Haemophilus influenzae* (5/100), *Haemophilus parahaemolyticus* (5/100), *Parvimonas micra* (5/100), *K. pneumoniae* (4/100), *S. aureus* (4/100), *Pseudomonas aeruginosa* (3/100), *Streptococcus pneumoniae* (3/100), and *A. defectiva* (2/100). The 10 most common bacterial species in control patients were *S. pneumoniae* (6/100), *M. tuberculosis* (5/100), *H. influenzae* (5/100), *Streptococcus constellatus* (5/100), *H. parahaemolyticus* (4/100), *P. micra* (3/100), *K. pneumoniae* (3/100), *S. aureus* (3/100), *P. aeruginosa* (2/100), and *Streptococcus retardans* (2/100). The prevalence of *A. baumannii* was significantly higher in patients with exacerbations (χ^2^ = 5.437, *P* = .02). The frequency of *A. baumannii, M. tuberculosis*, and *K. pneumoniae* in sputum samples from patients with acute exacerbation was 4% (4/100), 4% (4/100), and 3% (3/100), respectively. Intergroup differences in this frequency were not significant. The detection of viruses was significantly higher in BALF samples from patients with acute exacerbation (24% vs 9%) (χ^2^ = 6.954, *P* < .01). The 5 most commonly isolated viruses in the exacerbation group were human γ herpesvirus type 4 (16%), human β herpesvirus type 7 (7%), human α herpesvirus type 1 (6%), human β herpesvirus type 5 (3%), and human β herpesvirus type 6A (2%). The 5 most commonly detected viruses in controls were human β herpesvirus type 7 (5%), human γ herpesvirus type 4 (3%), human β herpesvirus type 5 (1%), human β herpesvirus type 6A (1%), and *Torque teno virus* (1%) (Fig. 4). The detection of human γ herpesvirus type 4 and human α herpesvirus type 1 was significantly higher in the acute exacerbation group (χ^2^ = 8.619 and χ^2^ = 5.437, *P* = .003 and *P* = .02, respectively). The prevalence of fungal species was marginally higher in the BALF of patients with acute exacerbation (22% vs 11%) (χ^2^ = 3.181, *P* = .074). Influenza virus was found in the sputum of one control patient.

**Figure 5.**
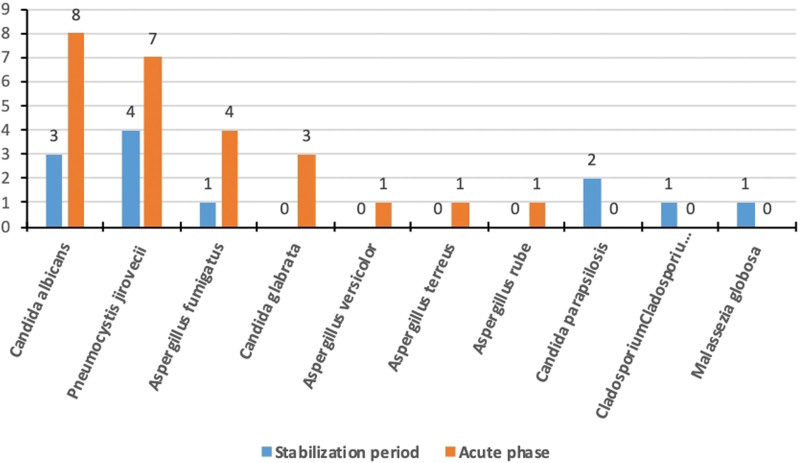
Frequency of detection of fungal species by metagenomic next-generation sequencing in patients with and without acute exacerbation of bronchiectasis.

**Figure 6.**
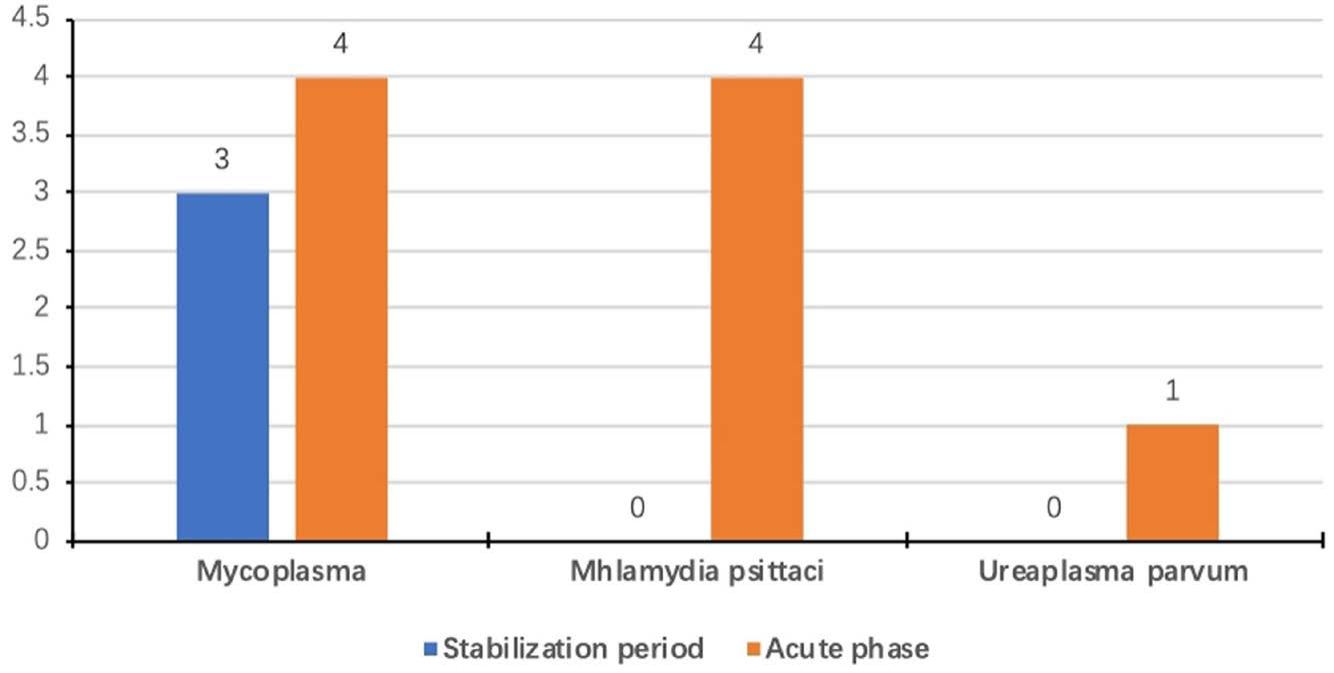
Frequency of detection of mycoplasma and *Chlamydia psittaci* by metagenomic next-generation sequencing in patients with and without acute exacerbation.

The 5 most common fungal species isolated from the sputum of patients with acute exacerbation were *C. albicans* (8%), *P. jirovecii* (7%), *A. fumigatus* (5%), and *Malassezia globosa* (2%). The 5 most frequent fungal species isolated from the sputum of control patients were *P. jiroveci*i (4%), *C. albicans* (3%), *Candida parapsilosis* (2%), and *A. fumigatus* (1%) (Fig. 5). *Mycoplasma* and *Chlamydia* were detected in 7 and 4 BALF samples, respectively, from the acute exacerbation group, and mycoplasma was detected in 4 BALF samples from control patients (Fig. 6). *Mycoplasma* was found in the sputum of one control patient. The rate of detection of *M. tuberculosis* in BALF samples from patients with and without exacerbation was 6.0% and 10.9%. The rate of detection of *M. tuberculosis* in sputum samples from these groups was 4% and 2%. The number of leukocytes and the serum levels of inflammatory markers (procalcitonin and C-reactive protein) were significantly higher in patients with acute exacerbation (Table 3).

**Table 3:** Number of leucocytes and the serum levels of inflammatory markers in patients with acute exacerbations of bronchiectasis and control patients.

Inflammatory markers	Acute exacerbation patients	Clinically stable patients	*t*	*P value*
Leucocytes (×10^9^/L)^a^	6.11 ± 1.29	8.66 ± 3.94	−4.474	<.001
Procalcitonin (ng/mL)^a^	0.03 ± 0.02	0.39 ± 0.87	−2.914	.005
IL-6 (ng/mL)^**a**^	4.43 ± 4.21	593.45 ± 3782.58	−1.032	.305
C-reactive protein (mg/L)^a^	4.26 ± 5.80	62.84 ± 76.01	−5.644	<.001

IL-6 = interleukin-6.

## 4. Discussion

The prevalence of bronchiectasis and exacerbations is increasing in China. The progression of bronchiectasis has been implicated with perturbation of the airway microbiome. No dedicated study has been performed using mNGS in bronchiectasis. We used mNGS technology to analyze the changes of microbiome in acute exacerbation and stable phase of bronchiectasis by comparing the results of alveolar lavage fluid in patients with acute exacerbation and stable phase. Our work highlights the versatility and usefulness of metagenomics in assessing variable component of the airway microbiome in bronchiectasis patients. Critically, we uncover that the frequency of detection of viruses was significantly higher in BALF samples from patients with acute exacerbation than in control patients, consistent with previous studies.^[6,7]^ The most commonly detected viruses in the BALF were human γ herpesvirus type 4, human β herpesvirus type 7, and human α herpesvirus type 1. Liu et al found that the most commonly isolated viruses in the lower respiratory tract were human herpesviruses, with human herpesvirus type 4 being the most common. The most commonly detected virus in sputum samples was the influenza virus. In turn, the most commonly isolated viruses in 119 patients with bronchiectasis in China were coronavirus, rhinovirus, and influenza.^[7]^ The most commonly detected viruses in 202 patients from Korea were influenza A virus and rhinovirus.^[6]^ The most prevalent virus in 217 patients with bronchiectasis in Asia and Europe was human parainfluenza virus 3.^[9]^ This inconsistency suggests that viral prevalence may vary by geographical region.^[10]^

The prevalence of fungal diseases in patients with bronchiectasis is increasing, and fungi can exacerbate the deterioration of lung function in such patients; however, the underlying mechanisms are unclear.^[11,12]^ The most commonly isolated fungal species in bronchiectasis patients are *C. albicans* (30%–45%) and *A. fumigatus* (7%–24%).^[11,13]^ Fungal colonization in the airways of patients with bronchiectasis may vary by geographical region and may be affected by the culture method and disease etiology.^[10,14]^ Our results underscore the need to pay attention to the screening and treatment of fungal infections in patients with acute exacerbations.^[15]^ Although the prevalence of tuberculosis (TB) has decreased in China in recent years, TB is a significant public health problem.^[16]^ In addition, TB is clustered in high-risk regions in China, especially Xinjiang.^[17–19]^ Although the prevalence of bronchiectasis due to tuberculosis has decreased because of increased vaccination and antituberculosis treatment, the frequency of *M. tuberculosis* in another cohort was high (11%),^[20]^ probably because the prevalence of bronchiectasis with *M. tuberculosis* is increasing.

Four cases of *C. psittaci* were detected in our samples. *C. psittaci* causes psittacosis, which is an emergent public health threat because of human-to-human transmission and the increased number of laboratory-confirmed cases reported in several countries. Furthermore, this disease is misdiagnosed because its symptoms are similar to those of other respiratory tract infections. Thus, all patients with respiratory diseases should be screened for *C. psittaci* infections. In addition, psittacosis should be included in the list of notifiable infectious diseases in China and elsewhere.^[21,22]^

Bronchiectasis is associated with bacterial colonization or infections in the lower respiratory tract. Our results showed that the most common bacterial species in the BALF of patients with acute exacerbation were *M. tuberculosis, H. influenzae*, and *S. pneumoniae*. Moreover, the number of gram-negative bacteria was significantly higher in these patients. The most common bacterial species in sputum were *M. tuberculosis, A. baumannii*, and *K. pneumoniae*. The frequency of *P. aeruginosa* was low, suggesting increased colonization by *H. influenzae*. In turn, previous studies have shown that the most prevalent bacterial species in the lower respiratory tract are *P. aeruginosa* and *H. influenzae*.^[23,24]^ In contrast, the most abundant pathogen in the lower respiratory tract of 136 inpatients with bronchiectasis in Peking Union Medical College Hospital was gram-negative bacilli, especially *P. aeruginosa*,^[25]^ suggesting that bacterial abundance varies depending on several factors, including geographical region and race.^[10,26]^

The results of the impact of the microbiome on clinical characteristics, including bronchiectasis progression, are inconsistent across studies. For instance, one study found that airway microbiome diversity and composition were significantly correlated with clinical markers of disease severity. In turn, another study observed that the microbiome did not change significantly in a 6-month period in patients with bronchiectasis, despite antibiotic therapy and changes in clinical status. Similarly, a longitudinal study found that the microbial community in sputum samples analyzed by 16S rRNA sequencing was relatively stable during a 16-year follow-up. Moreover, *H. influenzae* and *P. aeruginosa* had antagonistic effects in these samples. Another longitudinal study found that α and β diversity was poorly correlated with clinical parameters such as disease severity and duration.^[23,24,27,28]^

In summary, our results showed that the frequency of detection of viruses, fungi, bacteria, and *Mycoplasma* was higher in patients with acute exacerbation of bronchiectasis than in clinically stable patients. In addition, the most prevalent viruses in the acute exacerbation group were human γ herpesvirus type 4, human β herpesvirus type 7, and human α herpesvirus type 1. Specifically, the most commonly isolated fungal species were *C. albicans, P. jirovecii*, and *A. fumigatus*. Fungal infections are common in patients with bronchiectasis and are associated with faster deterioration of lung function. The most commonly detected bacterial species were *A. baumannii* and *H. influenzae*. The detection of *Mycoplasma* in our samples may reflect the increased prevalence of this pathogen in patients with bronchiectasis. In addition, the presence of *M. tuberculosis* in our samples reflects the high burden of tuberculosis in China, especially in Xinjiang. Tuberculosis is one of the main causes of bronchiectasis in China. *C. psittaci* was detected in 4 samples, underscoring the need to screen for this infectious agent in clinical practice. Most of our patients (55%) had mixed infections, indicating the need to target these infections in patients with acute exacerbation of bronchiectasis. The mechanisms underlying the effect of microbial interactions on disease progression should be further researched to increase the effectiveness of antibiotic therapy. Overall, this study showed that the airway microbiome of acute exacerbated bronchiectasis patients is more diverse than that of stable patients. mNGS has provided researchers with a better understanding of the changes in the airway microbiome in bronchiectasis patients.

One of the limitations of this study was the single-center design and small sample size, which may lead to analytical bias. Limitations of this study include the small sample size, which may not be representative of the entire population of bronchiectasis patients. Furthermore, the use of BALF as the sample source may not be reflective of the entire lung microbiome. Furthermore, the use of mNGS may not be able to detect all microbial species present in the sample. Finally, the study was conducted in a single center, which may limit the generalizability of the results. Furthermore, the Limitations section should discuss any potential implications of the study’s findings, such as the generalizability of the results or the potential for bias. Finally, the Limitations section should provide suggestions for future research that could address the limitations of the current study. Additionally, we just measured how many times a microorganism species was found in acute exacerbation patients versus clinically stable patients. In future studies, we will determine how much of each species is represented in each microbiome based on reads mapped or other quantitative metrics.

## Acknowledgment

We are grateful to all patients and their families.

## Author contributions

Writing—original draft: Dongmei Lu.

Conceptualization: Chenxi Li.

Writing—review & editing: Chenxi Li.

Data curation: Zhiwei Zhong, Maidina Abudouaini, Aynazar Amar.

Formal analysis: Chenxi Li, Zhiwei Zhong, Maidina Abudouaini, Aynazar Amar.

Funding acquisition: Dongmei Lu.

Investigation: Zhiwei Zhong, Maidina Abudouaini, Aynazar Amar.

Methodology: Dongmei Lu, Chenxi Li.

Supervision: Hongtao Wu, Xuemei Wei.

Validation: Dongmei Lu, Hongtao Wu, Xuemei Wei.
